# Tuning the plasmonic resonance in TiN refractory metal

**DOI:** 10.1038/s41598-024-55000-0

**Published:** 2024-04-04

**Authors:** Anchal Rana, Neeraj Kumar Sharma, Sambhunath Bera, Aditya Yadav, Govind Gupta, Abhimanyu Singh Rana

**Affiliations:** 1 Centre for Advanced Materials and Devices, School of Engineering and Technology, BML Munjal University, Sidhrawali, Gurugram, Haryana 122413 India; 2 CSIR-National Physical Laboratory, K.S. Krishnan Marg, New Delhi, 110012 India

**Keywords:** Materials for devices, Materials for optics, Nanoscale materials, Nanoscience and technology, Optics and photonics, Physics

## Abstract

Plasmonic coatings can absorb electromagnetic radiation from visible to far-infrared spectrum for the better performance of solar panels and energy saving smart windows. For these applications, it is important for these coatings to be as thin as possible and grown at lower temperatures on arbitrary substrates like glass, silicon, or flexible polymers. Here, we tune and investigate the plasmonic resonance of titanium nitride thin films in lower thicknesses regime varying from ~ 20 to 60 nm. High-quality crystalline thin films of route-mean-square roughness less than ~ 0.5 nm were grown on a glass substrate at temperature of ~ 200 °C with bias voltage of − 60 V using cathodic vacuum arc deposition. A local surface-enhanced-plasmonic-resonance was observed between 400 and 500 nm, which further shows a blueshift in plasmonic frequency in thicker films due to the increase in the carrier mobility. These results were combined with finite-difference-time-domain numerical analysis to understand the role of thicknesses and stoichiometry on the broadening of electromagnetic absorption.

## Introduction

Plasmonics play an important role in a wide range of applications in communication, medical, and energy harvesting devices^1–5^. Numerous efforts have been made for tuning the plasmonic absorption across the electromagnetic spectrum from visible to infrared range^6–8^. Among different materials, noble metals such as silver (Ag) and gold (Au) have been the most studied materials, as the absorption frequency lies in the visible and near-IR range, which can be further tuned with the size and shape of the nanoparticles^8^. However, their applicability is largely restricted at high temperatures, especially in solar-thermal applications^9^. The alternative is to use the refractory metals^9–11^. Transition metal nitrides have garnered significant attention due to their high melting points, excellent chemical stability, and superior mechanical properties to sustain harsh conditions^10–17^. Indeed, titanium nitride (TiN) is the most interesting material among other nitrides having high conductivities and local-surface-enhanced-plasmonic-resonance (LSPR) similar to Ag and Au^18–20^. Many researchers have explored TiN for plasmonic-based sensing, photothermal imaging, thermochromic windows, and energy conversion devices^12,17,21–27^. Particularly, to realize the application of plasmonic coatings for solar panels and smart windows, thin films of TiN should be as low as possible so that the enough radiation can be transmitted, and that to be grown at lower temperature to widen their scope for any arbitrary substrate material. However, it is a daunting task to control the desired plasmonic properties of non-noble metals and metal-nitrides due to the easier formation of oxides^5^. Therefore, vacuum-based techniques namely magnetron sputtering^28–30^, pulsed laser deposition (PLD)^31^, molecular beam epitaxy (MBE)^23^, transferred arc plasma^25^, plasma-enhanced atomic layer and chemical vapor depositions^32,33^ have been applied to get high crystalline TiN thin films showing plasmonic character. Most of these films were either deposited on crystalline substrates like Sapphire, MgO, and SiO_2_ at high temperatures or the thickness of the films were high enough to see any transmission spectra. Interestingly, Reddy et al.^28^ reported the temperature dependent plasmonic properties of TiN thin films of varying thicknesses (including low-thickness range) on sapphire substrates grown at high temperature (~ 850 °C) using sputtering. These films clearly showed superior plasmonic properties at high temperatures compared to noble metals^28,34^. Clearly, It is of interest to see whether the plasmonic properties can be retained in thinner films even if the films are grown at relatively lower temperatures^33,35^. Chang et al.^36^ were able to grow TiN thin films at low-temperature using sputtering on various substrates. However, the properties of these films were reported for higher thickness regime and the nanodisks were fabricated on Sapphire using electron beam lithography to obtain the transmission across the substrate^36^. Cathodic vacuum arc-deposition (CVA) has several advantage to grow highly metallic TiN thin films at lower temperatures^37–40^. However, CVA grown films at lower temperature are mostly amorphous and usually lead to the formation of macroparticles. Therefore, there are not many reports on the plasmonic behaviour on CVA deposited films. In contrast to earlier studies, the high crystalline TiN thin films with smooth surfaces have been obtained using CVA technique at ~ 200 °C by applying a negative substrate bias of − 60 V. The electron density determined by X-ray Reflectivity (XRR) of these films was found to be nearly comparable to that of bulk. Therefore, the plasmonic properties of these films matches with the films grown at high temperatures and thicker films using techniques like MBE, PLD and sputtering. We have observed that thinner films were highly stoichiometric which is critical factor for influencing the optical and plasmonic properties. For the first time, we have performed the finite-difference-time-domain analysis (FDTD) to model the optical and plasmonic properties to gain the further insights of the role of thickness and stoichiometry on these properties.

The optimizing the quality factor of SPP and LSPR in TiN films may become a crucial avenue for exploration. Strategies such as refining deposition techniques, exploring alternative materials or nanostructures, and tailoring the TiN film properties may help overcome these limitations. This optimization could unlock broader applications for TiN in practical scenarios where high-quality plasmonic effects are essential.

## Experimental

Titanium nitride (TiN) thin films were grown on well-cleaned barium-borosilicate 7059 high-grade glass substrates using a cathodic vacuum arc (CVA) deposition technique by evaporating a pure titanium metal target (75 mm diameter and ~ 99.999% purity) in the background nitrogen pressure of ~ 1 × 10^–3^ mbar. Prior to that, the base pressure better than ~ 5 × 10^–6^ mbar was achieved. The deposition was performed using an arc current of ~ 160A and a negative substrate bias of − 60 V. The deposition temperature was maintained at ~ 200 °C during the process. The deposition time was varied to obtain TiN films with different thicknesses (10 s, 20 s, and 30 s) named Sample A, B, and C respectively. The crystal structure and thickness of the films were analyzed by X-ray diffraction (XRD) and X-ray reflectivity (XRR) using a Panalytical XRD diffractometer with Cu Kα radiation at a wavelength of 1.54 Å. Atomic force microscopy (AFM) and Raman spectroscopy were performed using an integrated system by Witec (Alpha 300). Raman spectra were acquired using a green laser (532 nm) with a ~ 5 mW incident power. Optical transmission spectra were obtained using the UV–Vis spectrometer (LAMBDA 365, Perkin Elmer). X-ray photoelectron spectroscopy (XPS) was performed using Thermo Fisher (k Alpha) equipment. Ellipsometry was performed using M-2000U by J.A Woollen having a tungsten and deuterium lamp as an ultraviolet (UV) and visible light source. These measurements were carried out at an angle of 55° at room temperature. Photoluminescence (PL) and Time-Resolved-Photoluminescence (TRPL) measurements were carried out using an Edinburgh (FLS 980D2D2) setup. For PL and TRPL, the excitation wavelengths 244 nm and 266 nm were used, respectively.

## Result and discussion

### Crystallographic structure and surface analysis

**Figure 1:**
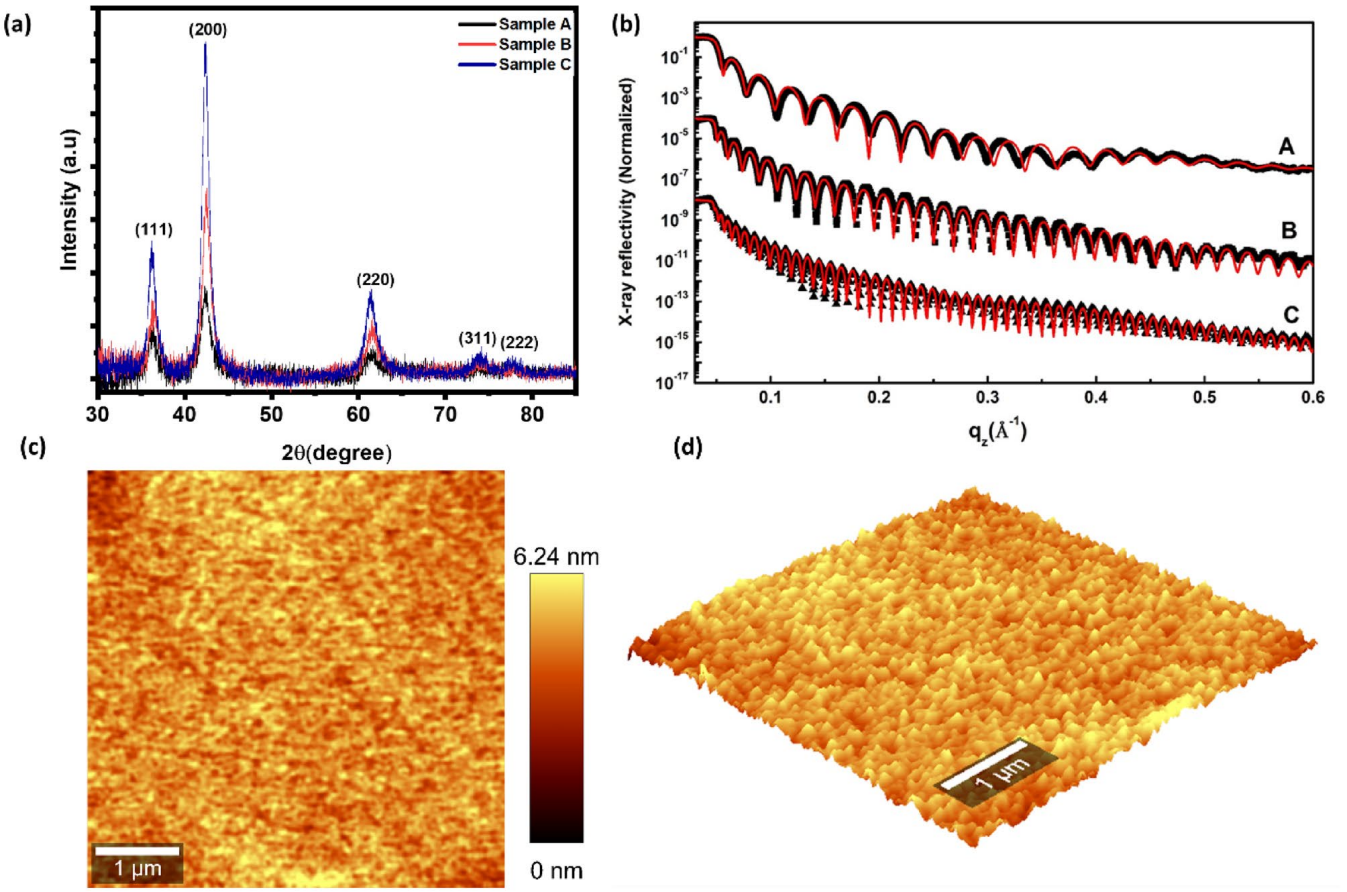
(**a**) XRD pattern (**b**) experimental specular XRR data (different black symbols) along with corresponding theoretical fitting (red solid curves) for the three TiN thin film samples. (**c**) AFM 2D topographic image (5 × 5 µm) with height colour scale bar of Sample B (**d**) corresponding AFM 3D image.

Figure 1a shows the X-ray diffraction (XRD) pattern of CVA-grown TiN films for different deposition times. The pattern exhibits strong peaks at ~ 36.2°, 42.3°, and 61.27°, corresponding to the crystal planes (111), (200), and (220) of TiN, respectively. This indicates that TiN possesses a cubic crystal structure. Additionally, peaks at ~ 74° and 77.8° can be attributed to the (311) and (222) planes, respectively^38^. Also, the observed peak positions are slightly shifted towards higher angles due to the presence of strain within the TiN crystal structure^41^. Moreover, the film thicknesses were determined using X-ray reflectivity (XRR). Figure 1b shows the XRR spectrum up to q_z_ = 0.6 Å^−1^ [where q_z_ $=\frac{4\Pi }{\lambda }{\text{sin}}\theta$] three TiN samples labeled as A, B, and C along with their respective best theoretical fit. The XRR pattern of all samples shows numerous oscillations, indicating very high-quality films. Below the critical angle, incident X-rays are completely reflected from the TiN film surface. As the angle of incidence reaches the critical angle, X-rays start to penetrate the TiN film, leading to a decrease in reflectivity (I/I_0_) above the critical angle. The electron density of the films was determined by fitting the critical angle region. Above the critical angle, a series of maxima and minima appear in the reflectivity pattern due to the constructive and destructive interference of the x-rays between the air/film and film/substrate interface. The period of oscillation in the reflectivity pattern is inversely proportional to the film thickness. The oscillation period is gradually decreasing from sample A to sample C, suggesting an increase in film thickness from sample A to sample C. We determined the electron density, thickness, and root-mean-square (RMS) roughness of the film from the fitting of the reflectivity data using Parratt formalism^42^. The XRR fitting results for the three samples are tabulated in Table 1. It was observed that all three samples have a low-density layer of thickness of approximately ~ 10 Å at the film surface due to surface oxidation. The calculated film thickness of sample A, sample B, & sample C is 207 Å, 333 Å, and 581 Å respectively. The electron density of the films was found 1.55 e^−^/Å^3^. Additionally, the mass density was derived from the electron density obtained from XRR fitting and found to be 5.43 gm/cm^3^, matching the bulk density of TiN. The roughness of all three films comes out to be very small (3 Å) indicating a very smooth surface. The surface topography and roughness were further checked by atomic force microscope (AFM) and the results are shown in Fig. 1c,d. The AFM images clearly show a smooth surface of these films with a dense microstructure. The surface RMS roughness is found to be ~ 1–3 Å which is consistent with the XRR measurements. This smooth surface with dense microstructure again indicates the high quality and uniformity of the TiN thin films.

**Table 1:** XRR fitting parameters of the three TiN thin film samples.

Layers		TiN	TiN-Oxide
Sample A	Electron density (e^−^/Å^3^)	1.55 ± 0.05	1.01 ± 0.05
	Film Thickness (Å)	196 ± 2	11 ± 2
	Roughness (Å)	3.0 ± 0.5	3.0 ± 0.5
Sample B	Electron density (e^−^/Å^3^)	1.55 ± 0.05	1.17 ± 0.05
	Film Thickness (Å)	322 ± 2	11 ± 2
	Roughness (Å)	5.0 ± 0.5	3.0 ± 0.5
Sample C	Electron density (e^−^/Å^3^)	1.55 ± 0.05	0.99 ± 0.05
	Film Thickness (Å)	569 ± 2	12 ± 2
	Roughness (Å)	4.0 ± 0.5	3.0 ± 0.5

### Chemical composition and stoichiometry

**Figure 2:**
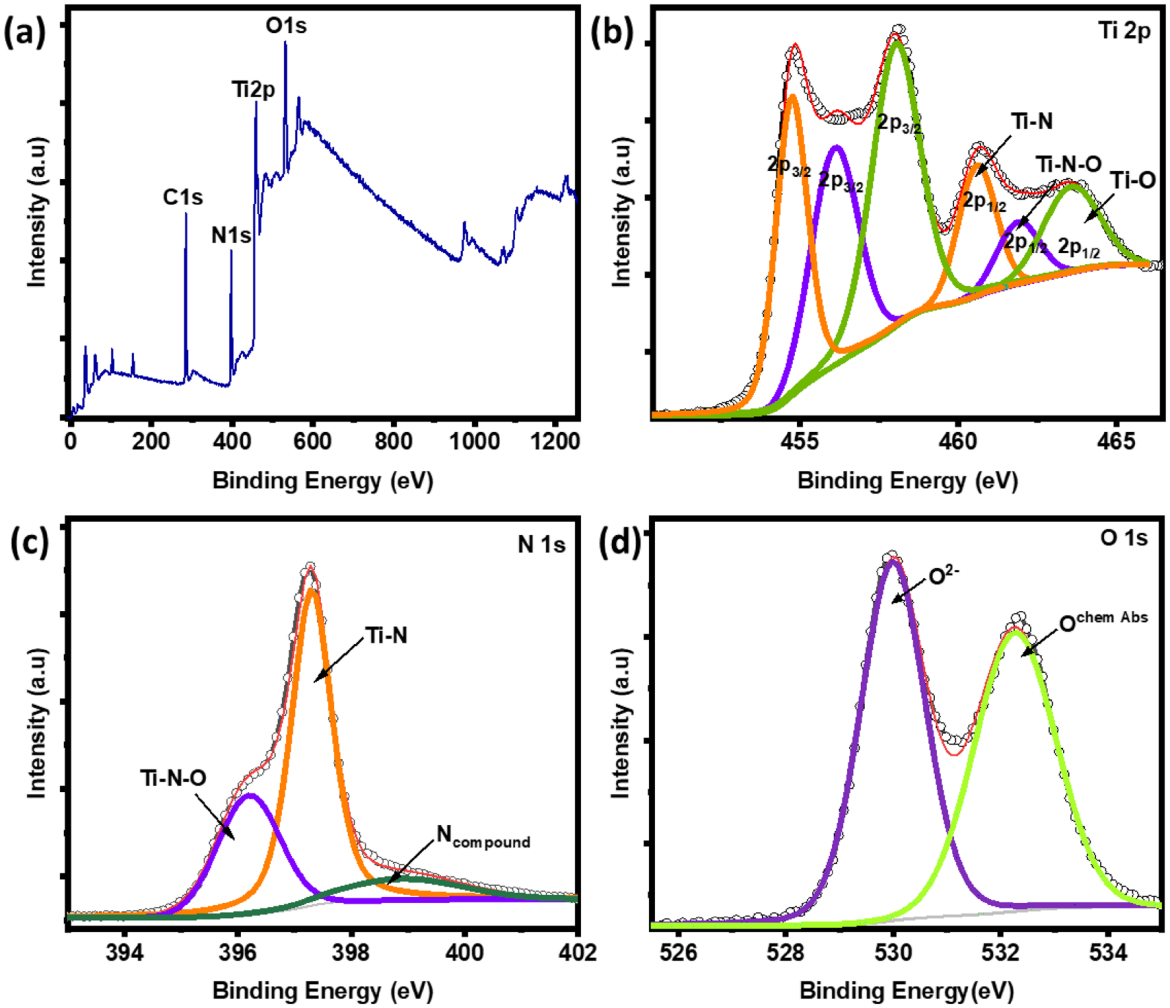
(**a**) XPS survey spectra (**b**–**d**) XPS spectra of core energy of Ti(2p), N(1s), and O(1s) of Sample B.

The chemical composition of these films was confirmed using X-ray spectroscopy (XPS) measurements. The XPS spectra in Fig. 2 (a-d) show the TiN film's core energy level of Ti 2p, N 1s, O 1s, and C 1s. The survey scan spectra clearly show the presence of these elements. Also, the survey shows the presence of oxygen in the TiN film, which was also confirmed by the XRR measurement that can be due to oxidation and the formation of a thin oxide/oxynitride layer on the TiN surface. The raw data was fitted by using Peak41 software [XPSPEAK Version 4.1 by Raymund Kwok (URL link: informer.com)] and is shown in Fig. 2. The Ti 2p orbital spectrum of TiN exhibits multiple peaks which show different Ti bonding states. The peaks at 454.78 eV (p_3/2_) and 460.58 eV (p_1/2_) correspond to Ti–N bonding, indicating the presence of TiN. The peaks at 456.18 eV (p_3/2_) and 461.98 eV (p_1/2_) in the spectra can be due to the formation of an oxide layer on the TiN thin film. These peaks indicate the presence of the + 4 oxidation state of titanium and suggest the formation of titanium dioxide (TiO_2_)^36^. In addition, the presence of an intermediate state between TiO_2_ and TiN, known as titanium oxynitride (TiN_x_O_y_), was observed in Ti spectra. This is shown by the peak at 458.08 eV (p_3/2_) and 463.58 eV (p_1/2_). In N 1 s spectrum, three peaks at 396.18 eV, 397.28 eV, and 398.98 eV were observed. The highest peak is associated with the TiN phase, indicating the presence of nitrogen in the TiN film. The second peak at 397.28 eV corresponds to TiN_x_O_y_. The third peak at 398.98 eV suggests the presence of nitrogen compounds in the film, which could arise from other nitrogen-containing species or impurities^21^. The O 1s spectrum exhibits two peaks at 529.98 eV and 532.28 eV, which can be attributed to O^2+^ and chemically absorbed oxygen (O_chem Abs_) on the surface of TiN^43^.

### Raman analysis for crystallographic defects

**Figure 3:**
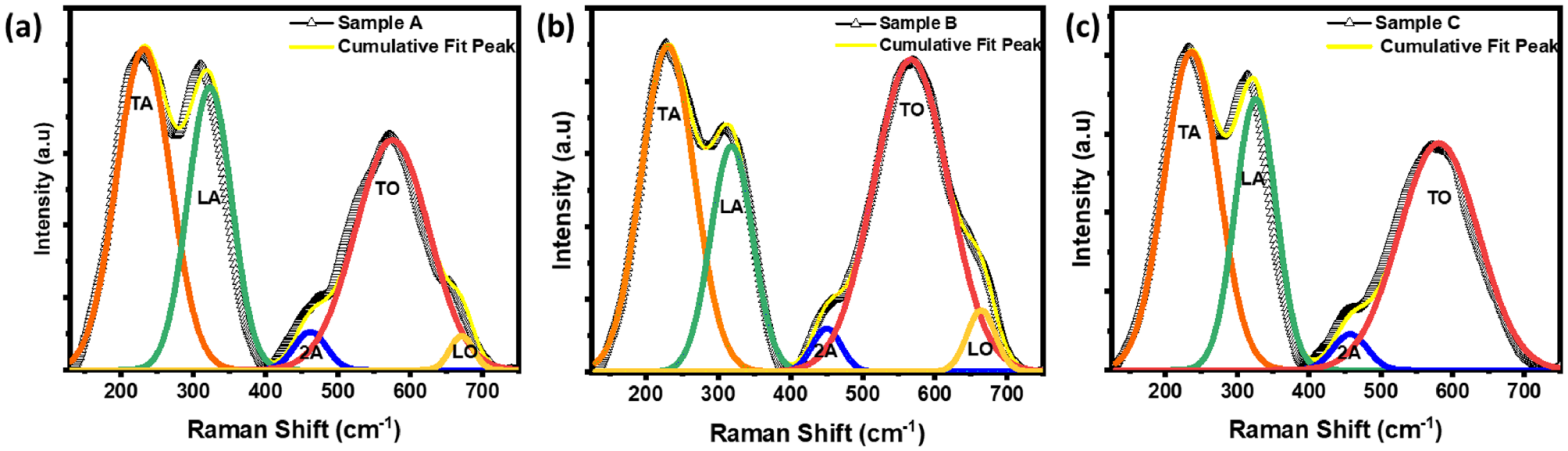
(**a**–**c**): Raman spectra of TiN thin films of samples A, B, C respectively.

Figure 3a–c shows the Raman spectrum of TiN thin films. Raman spectra is important to investigate the properties of TiN thin films. Especially, the bulk TiN lacks first-order Raman scattering due to its octahedral (Oh) symmetry but the defects or vacancies within the TiN lattice can induce the Raman scattering^44^. However, it is challenging to obtain the Raman spectra as the high reflectivity of the samples limits the penetration depth of incident light due to its high metallic nature. Overcoming these challenges requires specialized conditions^45^. A high-power laser is often necessary to achieve a high-resolution spectrum with a good signal-to-noise ratio to enhance the Raman signal. However, the use of high-power lasers can potentially lead to oxidation and the formation of TiO_2_ on the TiN films. The optimization of experimental conditions is crucial to achieving reliable Raman measurements while minimizing the effects of oxidation. The spectra were acquired using low laser power to minimize the effects of oxidation. The curves were fitted with a Gaussian function using peakfit software. The spectrum of these samples reveals first-order phonon frequencies at ~ 227–230 cm^−1^, 316–318 cm^−1^, 567–575 cm^−1^, and 663–668 cm^−1^, corresponding to the transverse acoustic (TA), longitudinal acoustic (LA), transverse optical (TO), and longitudinal optical (LO) modes, respectively, of cubic nanocrystalline TiN. Additionally, a second-order acoustic mode is observed at ~ 449 cm^−1^^46,47^. The first-order acoustic modes arise from vibrations of Ti atoms near N vacancies, while vibrations of N atoms adjacent to Ti vacancies contribute to the first-order optical bands. The presence of low-intense second-order phonon modes confirms the existence of point defects in the TiN film. Overall, the observation of first-order acoustic and optical modes confirms the presence of Ti and N vacancies in the TiN lattice.

### Optical and plasmonic properties

**Figure 4:**
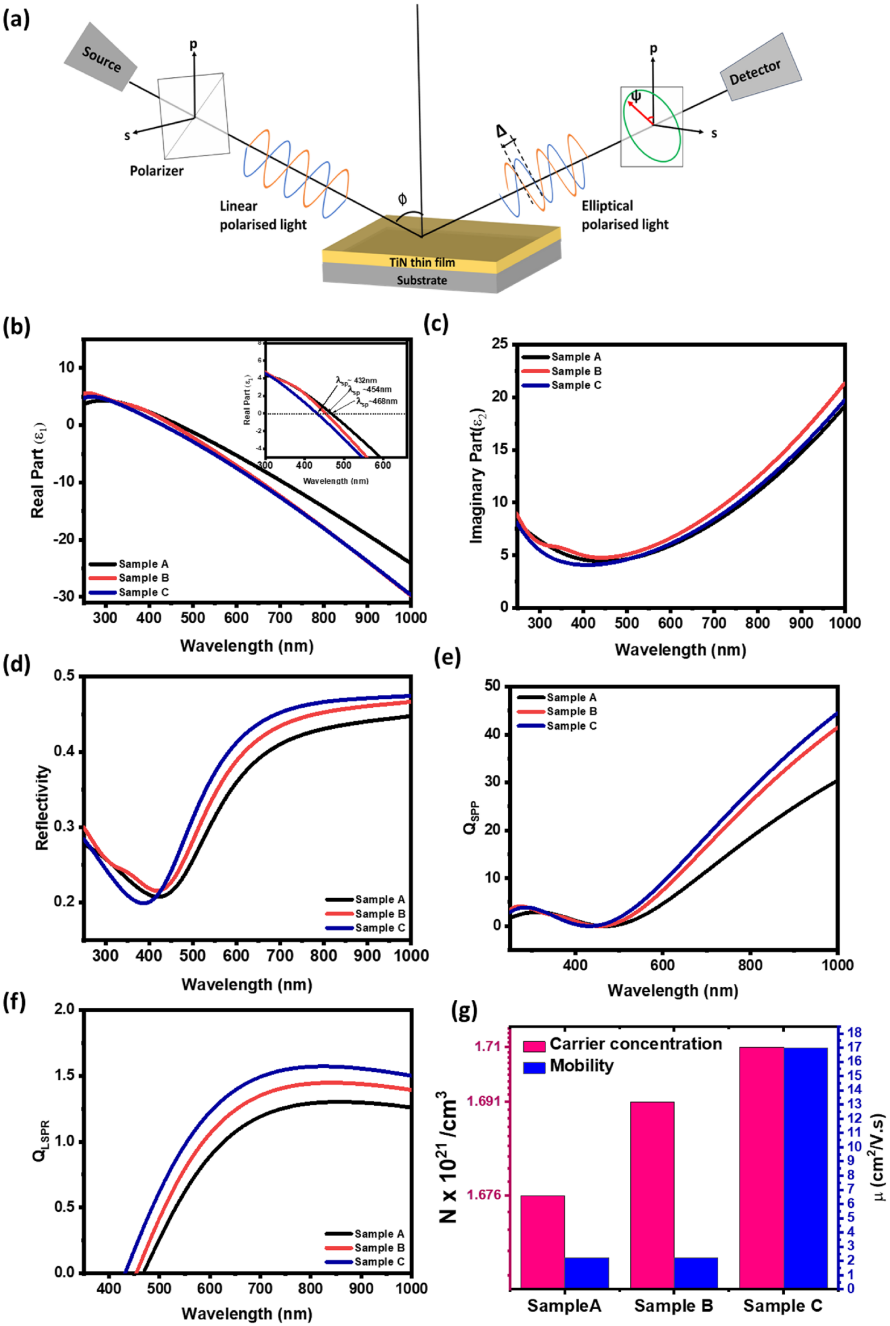
(**a**) Schematic diagrams of ellipsometry set-up (**b**, **c**) Real and imaginary part of the dielectric function with wavelength measured at an angle of 55°, (**d**) reflectivity calculated from the refractive index (n) and extinction coefficient (k), and (**e**, **f**) quality factor for SPP and LSPR of all three samples. (**g**) The Hall carrier concentration and mobilities of all three samples.

The optical properties of these films were investigated using ellipsometry from ultraviolet (UV) to infrared (IR) range and the results are shown in Fig. 4. Figure 4a illustrates the schematic picture of the experimental design. The values of psi ($\psi$) and delta (Δ) were measured at different wavelengths, and the dielectric constants were derived from the acquired ellipsometry quantities using the given mathematical expression as

1 $$\langle \varepsilon \rangle ={{\text{sin}}}^{2}\varnothing \left[1+{{\text{tan}}}^{2}\varnothing \left(\frac{1-\rho }{1+\rho }\right)\right]$$

2 $$\langle \varepsilon \rangle ={\varepsilon }_{1}+{\varepsilon }_{2}$$

where ε_1_and ε_2_ is the real and imaginary part of the dielectric constant, $\varnothing$ is the angle of incidence, and $\rho =\frac{{R}_{P}}{{R}_{s}}={\text{tan}}\psi {e}^{i\Delta }$ is the ratio of the total reflection coefficient^48^. The dielectric function of TiN was determined by fitting ellipsometry data with a Drude-Lorentz oscillator model and the quality of fitting was ensured by minimizing the mean-squared-error (MSE) value^49^, which was less than 3 in this case. The model includes a Drude oscillator, providing information about the behavior of free d-band conduction electrons, and Lorentz oscillators, which explain the inter- and intraband transitions in the material^50^. Subsequently, the ε_1_ and ε_2_ were calculated based on the refractive index (n) and extinction coefficient (k), given by

3 $${\varepsilon }_{1}={n}^{2}-{k}^{2}$$

4 $${\varepsilon }_{2}=2nk$$

The ε_1_ and ε_2_ of the dielectric function of TiN thin films are shown in Fig. 4b,c. The real part of TiN thin films exhibits an increase from positive to more negative values as a function of wavelengths. This behavior indicates the presence of local-surface-enhanced-plasmonic-resonance (LSPR)^23^. The crossover plasma energy, *Esp* = *hc*/*λ*_*sp*_, where h is Plancks constant, c is the speed of light, and *λ*_*sp*_ is wavelength, can be calculated at ε_1_ = 0, known as the epsilon-near-zero (ENZ) point^51^. The graph shows that the real part of TiN thin films becomes more negative as the thickness of these films increases. The observed LPSR wavelength exhibits a blue shift with increasing in thicker film. The plasma energy (E_sp_) values are 2.64 eV, 2.73 eV, and 2.87 eV for TiN films for thicknesses ~ 20 nm, 30 nm, and 60 nm, respectively [see inset in Fig. 4b]. The dielectric function's imaginary part (ε_2_) for TiN thin films is shown in Fig. 4c. The hump observed in sample B within the wavelength range of 300–500 nm again indicates strong absorption of light in this region, likely due to inter- and intraband transitions occurring within the material^23^. On the other hand, sample A exhibits lower ε_2_ values in the near-infrared (NIR) region compared to other samples, suggesting reduced light absorption and energy dissipation. The total reflectivity (R) of the sample was calculated using the following expression as

5 $$R=\frac{{\left(n-1\right)}^{2}+{k}^{2}}{{\left(n+1\right)}^{2}+{k}^{2}}$$

The reflectivity versus wavelength of TiN samples is shown in Fig. 4d. The reflectivity behavior of TiN thin films typically follows a trend where it is higher in the near-infrared (NIR) region and drops as the wavelength decreases between 400 and 500 nm wavelength range. In the NIR region (near-infrared), TiN thin films typically exhibit relatively high reflectivity, which is characteristic of their metallic behavior. However, as the wavelength decreases towards the visible and UV region, the reflectivity of TiN thin films starts to decrease, and a minimum in the reflectivity spectrum was observed. The reflectivity dip at ~ 2.9–3.2 eV is associated with a plasmonic resonance^52^. As the optical and plasmonic properties of TiN thin films are determined by the real and imaginary parts of the dielectric function. However, plasmonic properties can provide further information on surface-plasmon-polaritons (SPPs) and LSPRs within the material by explaining the dielectric function quality expressed as^53^

6 $${Q}_{SPP}=\frac{{\left({\varepsilon }_{1}\right)}^{2}}{{\varepsilon }_{2}} \, \text{(For SPP)}$$

7 $${Q}_{LSPR}=\frac{-{\varepsilon }_{1}}{{\varepsilon }_{2}} \, \text{(For LSPR)}$$

Figure 4e,f shows a consistent trend in both Q_*SPP*_ (Quality factor for SPP) and Q_*LSPR*_ (Quality factor for LSPR). Sample C exhibits the highest Q_*SPP*_ and Q_*LSPR*_ values, followed by samples B and A, respectively. The higher Q_*SPP*_ and Q_*LSPR*_ values in the samples indicate stronger and more well-defined plasmonic resonances in these films. Compared to noble metals such as Ag and Au, the TiN films exhibit a lower quality factor for Surface Plasmon Polaritons (SPP) and Localized Surface Plasmon Resonance (LSPR). This lower quality factor may present a limitation for certain practical applications. While TiN is valued for its durability and compatibility, the observed differences in quality factor could impact its effectiveness in plasmonic applications. The blue shift in the LSPR frequency is also consistent with our Hall measurement results [see Fig. 4g]. The plasma frequency can also be approximated by the transport properties using the relationship ${\omega }_{sp}=\sqrt{\frac{N{e}^{2}}{{\varepsilon }_{0}{m}^{*}}}$, where *N*, m*, e, and *ε*_0_ are carrier concentration, effective mass, electron charge, and vacuum permittivity respectively.

**Figure 5:**
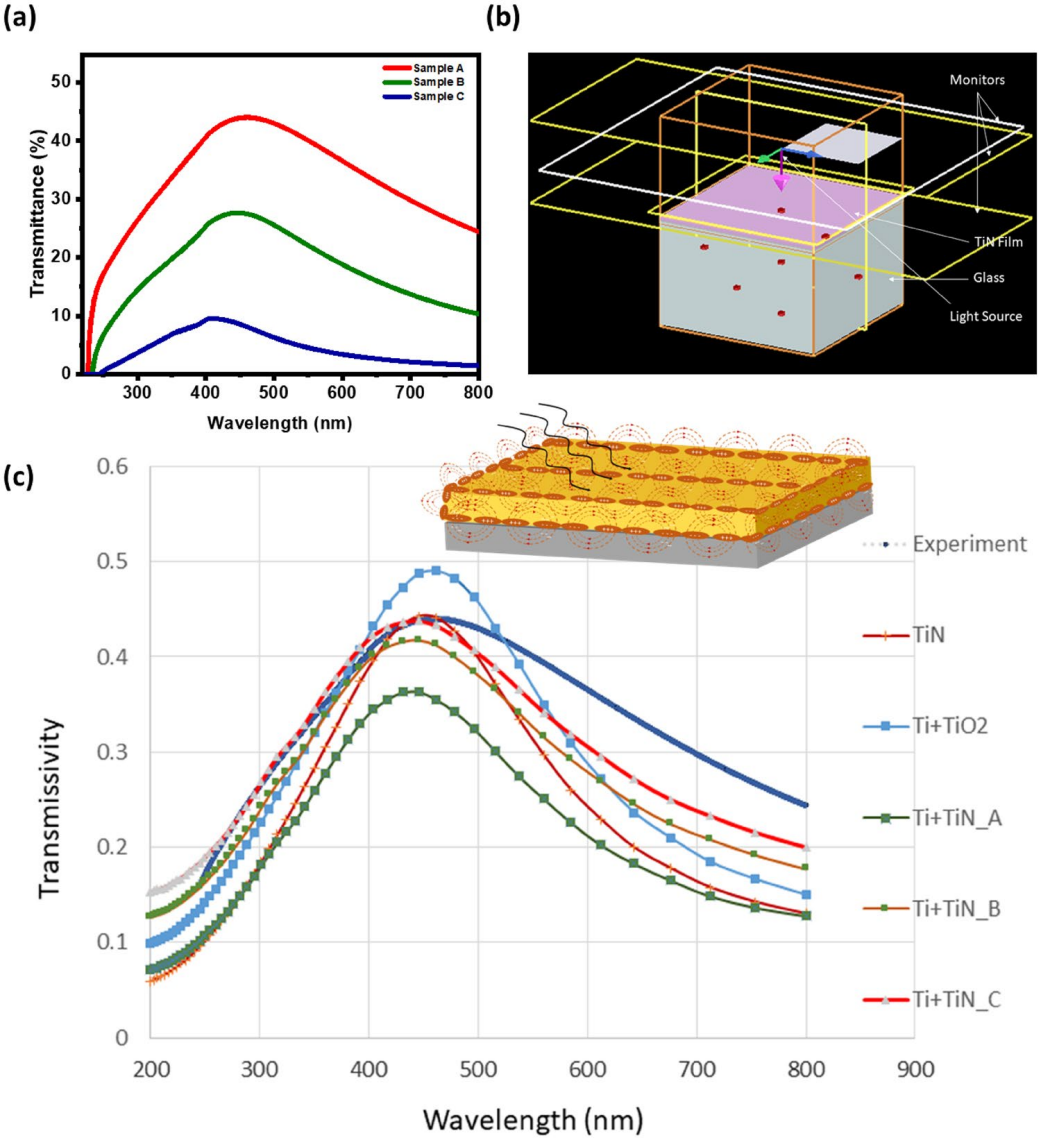
(**a**) The experimental transmission versus wavelength curve of TiN thin films on glass substrate of three different thicknesses. (**b**, **c**) FDTD analysis, (**b**) the image showing modelled light source and sample (TiN thin film coated on glass substrate), (**c**) the plots of transmission data obtained from FDTD simulations for different models, the inset shows the schematic plasmons interference between different interfaces.

To see the overall absorption spectra for any practical application, these films were measured using UV–visible spectroscopy in transmission mode, where the light source is perpendicular to the sample, a usual case in any solar-thermal device. Figure 5a shows the transmission versus wavelength curve of TiN thin films of three different thicknesses on the glass substrate. As expected, the overall transmission reduces as the thickness of these films increases. Also, a broad peak is observed between 400 and 500 nm wavelengths. TiN has a reported bandgap of ~ 3.35–3.5 eV (between 370 and 354 nm wavelengths), which is clearly not matching our transmission curve showing an absorption from visible to IR range. The peak value in sample C shows a blueshift as also observed in reflectivity measurements. This is against the understanding of bandgap shift as the dimensions reduce, usually due to quantum confinement and confirms the role of plasmons. Although, the role of surface plasmon is complex to understand when the source is perpendicular to sample surface. The transmission spectra are mostly dominated by the bulk plasmons and different interferences occurring between various plasmonic modes cause by different nanoscopic interfaces (having different dielectric constants), namely TiN–Glass, Ti–TiN, TiO_2_–TiN, Ti–TiO_2_, Ti–Glass.

### Finite-difference time-domain (FDTD) analysis

To gain insight into the cause of absorption broadening in TiN thin films and different role of these interfaces, we have performed the finite-difference-time-domain (FDTD) analysis of these films using ANSYS-Lumerical commercial software [ANSYS Lumerical 2023, Trial License (URL link: https://www.lumerical.com/downloads/customer/)] based on the structural and chemical inputs obtained experimentally in Figs. 1, 2, and 3. The FDTD results are shown in Fig. 5b,c.

#### Geometric modelling and simulation parameters

The glass substrate with TiN thin film was modelled using a 1000 nm square cross section. The substrate of a thickness of 500 nm and the thin film of variable thickness was modelled (see Fig. 5b). A plane wave source with a wavelength range between 200 to 800 nm was used to simulate UV–Visible spectrum in Fig. 5a. A plane wave source simulates a wave with parallel wave fronts where the phase is uniform across each wave front. Beams that are incident on a periodic structure with the spot size of the beam much larger than the period of the device can be best modelled using a plane wave source.

#### Mesh and boundary conditions

FDTD methos require the domain to be discretized and small elements and to calculate the electric and magnetic fields at each mesh points by applying the appropriate boundary conditions. An extremely fine mesh increases the computational cost, while the coarse mesh cannot correctly capture the transmission response, and therefore a mesh convergence study was conducted to obtain the optimum mesh element size. A nonuniform mesh with a minimum element size of 0.25 nm and a maximum element size of 2 nm was used for simulations. Out of the different type of boundary conditions used for FDTD analysis, the periodic boundary condition is most useful in the present case due to the symmetricity of both the fields and the structure. In order to reduce the computational cost, only a quarter of geometry was modelled by applying the symmetric boundary conditions in the x–y plane. Perfectly Matched Layer (PML) boundary condition were applied along z axis to measure the absorbed fraction of incident light. The refractive index data reported by Palik et al.^54^ for glass substrate, titanium nitride and titanium used in the simulation are provided in Supplementary Figure S1. The frequency-domain profile and power monitors were used at different locations in order to record the transmission spectrum of waves. A parametric analysis was conducted using FDTD method and different models were generated for the analysis (see Table 2). From the experimental analysis presented in Section “Chemical composition and stoichiometry” and Section “Raman analysis for crystallographic defects” in this manuscript, the presence of TiO_2_ and Ti is indicated. In order to investigate this further, varying nanostructure of TiN film with Ti and TiO_2_ constituents: layered and particulate, and thickness (from experiments thickness from 20 to 30 nm): 28 nm, 23 nm and 21 nm were generated. These models were simulated, and the transmission response of these models were compared with the experimental results.

**Table 2:** Different composition used to simulate the experimental results using FDTD.

FDTD Model	
TiN	28 nm TiN
Ti+TiO_2_	5 nm Titanium dioxide and 28 nm TiN
Ti+TiN_A	5 nm Titanium and 28 nm TiN
Ti+TiN_B	Ti–TiN Composite: Titanium particles reinforced in 23 nm TiN thickness film
Ti+TiN_C	Ti–TiN Composite: Titanium particles reinforced in 21 nm TiN thickness film

#### Simulation results from FDTD analysis

The simulated values of transmission response were compared with the experimental graph (see Fig. 5c). The transmission response of different nanostructures obtained from FDTD simulations has been compared with the experimental values. It can be observed that in case of pure titanium nitride thin film model, a peak of 0.438 of transmission response is obtained at 445 nm wavelength which is in good agreement with the experimental peak value. However, the pre and post peak value, the simulated values of transmissivity don’t match with the experimental values. As suggested from XRR and XPS result, there is an oxidation of unbonded titanium at surface and therefore a FDTD model with 5 nm layer at top of TiN film is modelled and the transmission response is simulated. It can be seen that the pre-peak values of Ti+TiO_2_ values are in better agreement with the experimental curve. However, a peak of transmissivity of 0.49 is obtained at 461 nm for this model, which is considerably higher that the experimental peak value. As observed in the experimental analysis section, the presence of Ti–TiN interfaces formed due to the nitrogen deficiency, which was also observed in our previous reported work^41^. Therefore, three more models of Ti/TiN having different nanostructures: Ti + TiN_A - 5 nm Ti layer above 28 nm TiN film, Ti+TiN_B–Ti particles reinforced in 23 nm TiN matrix composite and Ti+TiN_C–Ti particles reinforced in 21 nm TiN matrix composite, were further analysed. It can be observed that the layered nanostructure model in case of Ti+TiN_A predict considerably lower peak comparing to the experimental value. The composite models in which Ti particles reinforced in TiN film are in much better agreement with the experimental values. It can be observed that the pure TiN curve has a sharp peak while the presence of Ti particles, due to nitrogen deficiency can cause the broadening of transmission curve due to the interference between different Plasmonics occurring at these interfaces [see the inset of Fig. 5c]. It can be understood that the Ti reinforced TiN nanostructure will result into much more interfaces than that of layered nanostructure and therefore, has been resulted into higher transmission.

### Photoluminescence (PL) and time-resolved-PL (TRPL) analysis

**Figure 6:**
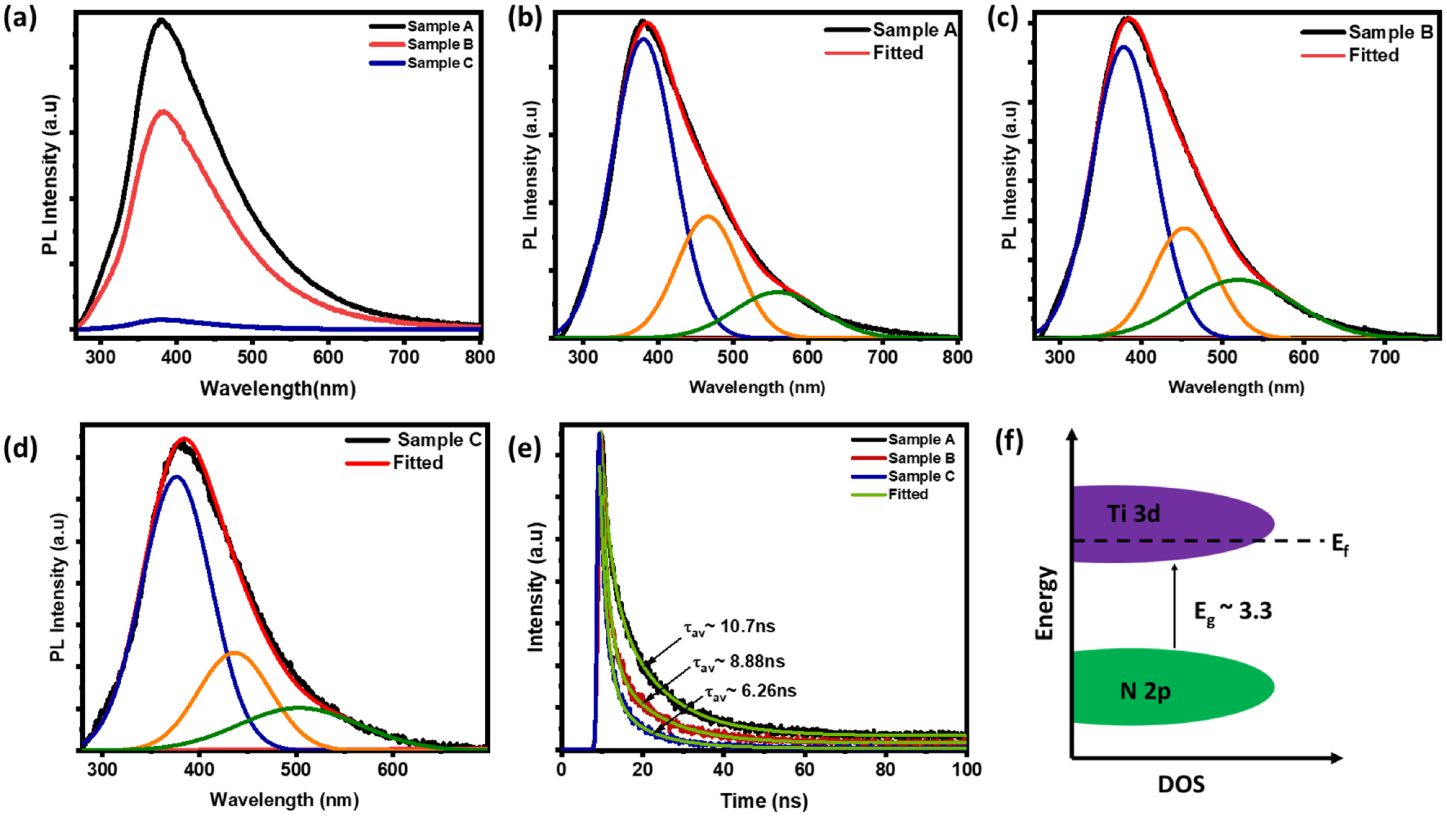
(**a**) PL spectra of Sample A, B and C, (**b**–**d**) the deconvoluted peaks, (**e**) TRPL spectra of Sample A, B, and C respectively and (**f**) schematic of energy band diagram of TiN.

Finally, to confirm electronic absorption mechanism and different electronic transitions^55^, the photoluminescence (PL) and time-resolved-PL (TRPL) measurements were performed on these samples, and the results are shown in Fig. 6a–e. A broad peak was observed in the UV–Visible-NIR region, with decreasing PL intensity with thickness. The high PL intensity in sample A suggests higher radiative recombination over non-radiative due to the fewer defects in thinner films as consistent with the transmission discussion. The broad peak in the PL spectrum was deconvoluted into three peaks using Gaussian fitting. The highest peak around ~ 380 nm for sample A, ~ 379 nm for sample B, and ~ 377 nm for sample C, correspond to band-edge transition between the conduction band of Ti and the valence band of N [see Fig. 6f]^56^. The second set of peaks at ~ 466 nm, ~ 453 nm, and ~ 436 nm are attributed to charge transfer excitations. The observed peak in the samples matches with the unscreened plasma wavelength determined by ellipsometry. The third shoulder peaks in the PL spectra at ~ 560 nm, ~ 520 nm, and ~ 503 nm can be associated with recombination of surface states due to oxide layer^57^. TRPL can further gives carriers decay time, and the results are shown in Fig. 6e for all three thickness. The decay time of charge carriers can be fitted with the binominal function using the formula given below:

8 $${\tau }_{av}=\frac{{A}_{1}{\tau }_{1}^{2}+{A}_{2}{\tau }_{2}^{2}}{{A}_{1}{\tau }_{1}+{A}_{2}{\tau }_{2}}$$

The fitting parameters are given in Table 3. The TRPL results revealed two distinct decay components (τ_1_ and τ_2_), corresponding to nonradiative recombination and radiative recombination, respectively. Nonradiative recombination is associated with the presence of defects in the material, while radiative recombination provides insights into the recombination of charge carriers^58^. The average decay time ~ 10.7 ns, 8.88 ns, and 6.26 ns for samples A, B, and C respectively. The shorter lifetime of carriers could be due to increase in the nitrogen vacancies. These results further confirming the tuning of Plasmonic broadening through defect formation by increasing the film thicknesses.

**Table 3:** TRPL fitting parameters of all three samples.

Sample Name	A_1_	A_2_	τ_1_ (ns)	τ_2_ (ns)	τ_av_(ns)
Sample A	677.679 ± 17.2963	761.272 ± 14.4945	2.357 ± 0.1074	12.13 ± 0.1555	10.7
Sample B	1100.708 ± 16.9259	440.039 ± 8.6126	1.797 ± 0.0438	11.62 ± 0.1634	8.88
Sample C	1094.783 ± 15.7334	305.885 ± 8.5463	1.77 ± 0.03928	9.316 ± 0.1494	6.26

## Conclusion

In conclusion, this research clearly demonstrates and provide deeper insights of the tunability of the plasmonic frequency in high-quality CVA deposited TiN thin films grown at lower temperature through the manipulation of thickness. The electron density of these films was found to be nearly comparable to that of bulk. There is clear a blueshift of plasmonic frequency as the film thickness decreases due to reduced effective mass. Also, the transmission spectra show a broad absorption from visible to IR range due to interference in plasmonic modes cause by different nanoscopic interface due to the presence of substrate and defects. These findings significantly contribute to a deeper understanding of commercially viable CVA-grown TiN refractory metal, particularly on glasses at lower temperatures for plasmonic based applications. However, recognizing the complexities of plasmonic interactions, it is crucial to approach these findings with a nuanced perspective. Future research could focus on more detailed investigations, delving into specific mechanisms and factors influencing plasmonic behavior in TiN thin films. Additionally, the enhancement of the quality factor of Surface Plasmon Polaritons (SPP) and Localized Surface Plasmon Resonance (LSPR) in TiN films through advanced deposition techniques or innovative material modifications may further broaden its practical applicability in the future.

### Supplementary Information

Supplementary Information.

## Data Availability

All data generated or analysed during this study are included in this published article and its supplementary information files.
