# Supplementary material for: Tuning the plasmonic resonance in TiN refractory metal
Source: Sci Rep. 2024 Apr 4;14:7905. doi: 10.1038/s41598-024-55000-0 (PMC10991307; doi:10.1038/s41598-024-55000-0)
Supplement: Supplementary file 1 — Supplementary Information. [file 41598_2024_55000_MOESM1_ESM.docx]

**Supplementary**

**Tuning the plasmonic bandwidth in TiN refractory metal**

Anchal Rana^1^, Neeraj Kumar Sharma^1^, Sambhunath Bera^1^, Aditya Yadav^2^, Govind Gupta^2^, Abhimanyu Singh Rana^1^*

^1^Centre for Advanced Materials and Devices, School of Engineering and Technology, BML Munjal University, Sidhrawali, Gurugram-122413, Haryana, India

^2^CSIR-National Physical Laboratory, K.S. Krishnan Marg, New Delhi, 110012, India

[rana.abhimanyu@gmail.com](mailto:rana.abhimanyu@gmail.com)

| 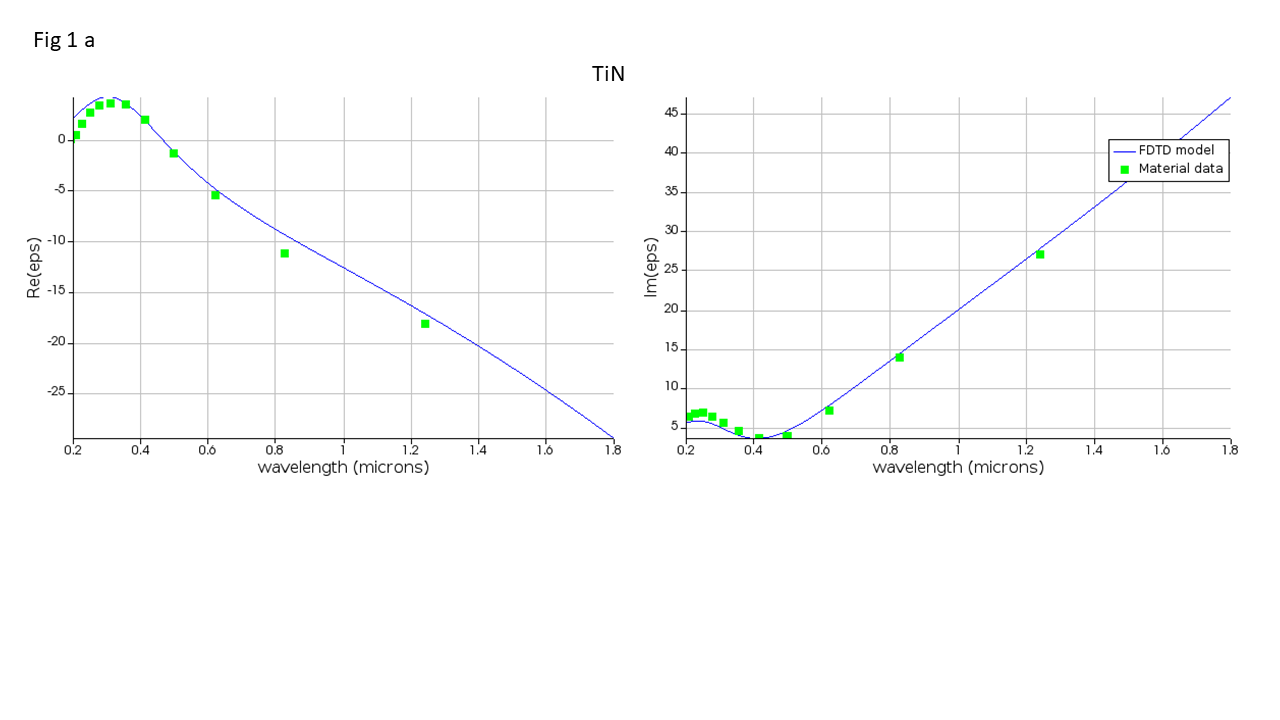**(a)** |
| --- |
| 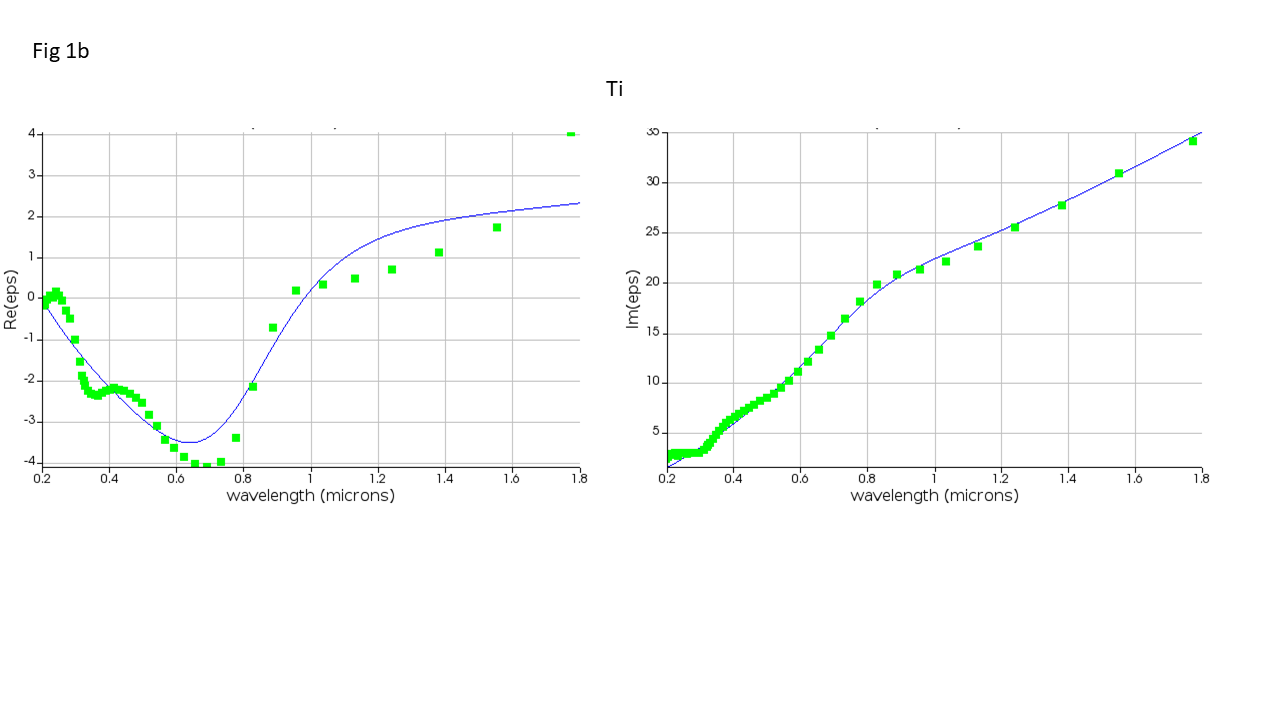**(b)** |
| 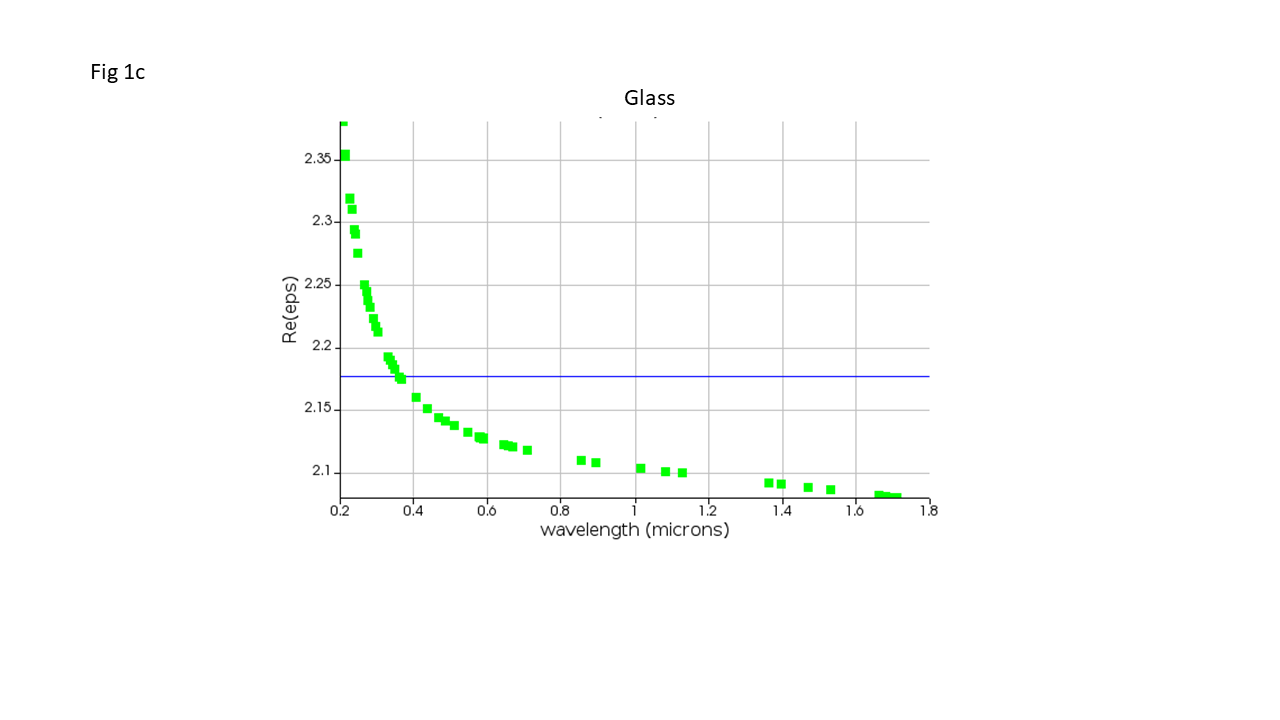**(c)** |

Figure S1: Wavelength dependent refractive index data used in simulation for Ti, TiN and glass
